# Development of a Just-in-Time Adaptive mHealth Intervention for Insomnia: Usability Study

**DOI:** 10.2196/humanfactors.8905

**Published:** 2018-05-17

**Authors:** I Wayan Pulantara, Bambang Parmanto, Anne Germain

**Affiliations:** ^1^ Health and Rehabilitation Informatics Laboratory Department of Health Information Management University of Pittsburgh Pittsburgh, PA United States; ^2^ Sleep and Chronobiology Laboratories Department of Psychiatry University of Pittsburgh Pittsburgh, PA United States

**Keywords:** Just-in-Time Adaptive Intervention, JITAI, mobile health, mHealth, sleep, insomnia, usability, smartphone, iREST

## Abstract

**Background:**

Healthy sleep is a fundamental component of physical and brain health. Insomnia, however, is a prevalent sleep disorder that compromises functioning, productivity, and health. Therefore, developing efficient treatment delivery methods for insomnia can have significant societal and personal health impacts. Cognitive behavioral therapy for insomnia (CBTI) is the recommended first-line treatment of insomnia but access is currently limited for patients, since treatment must occur in specialty sleep clinics, which suffer from an insufficient number of trained clinicians. Smartphone-based interventions offer a promising means for improving the delivery of CBTI. Furthermore, novel features such as real-time monitoring and assessment, personalization, dynamic adaptations of the intervention, and context awareness can enhance treatment personalization and effectiveness, and reduce associated costs. Ultimately, this “Just in Time Adaptive Intervention” for insomnia—an intervention approach that is acceptable to patients and clinicians, and is based on mobile health (mHealth) platform and tools—can significantly improve patient access and clinician delivery of evidence-based insomnia treatments.

**Objective:**

This study aims to develop and assess the usability of a Just in Time Adaptive Intervention application platform called iREST (“interactive Resilience Enhancing Sleep Tactics”) for use in behavioral insomnia interventions. iREST can be used by both patients and clinicians.

**Methods:**

The development of iREST was based on the Iterative and Incremental Development software development model. Requirement analysis was based on the case study’s description, workflow and needs, clinician inputs, and a previously conducted BBTI military study/implementation of the Just in Time Adaptive Intervention architecture. To evaluate the usability of the iREST mHealth tool, a pilot usability study was conducted. Additionally, this study explores the feasibility of using an off-the-shelf wearable device to supplement the subjective assessment of patient sleep patterns.

**Results:**

The iREST app was developed from the mobile logical architecture of Just in Time Adaptive Intervention. It consists of a cross-platform smartphone app, a clinician portal, and secure 2-way communications platform between the app and the portal. The usability study comprised 19 Active Duty Service Members and Veterans between the ages of 18 and 60. Descriptive statistics based on in-app questionnaires indicate that on average, 12 (mean 12.23, SD 8.96) unique devices accessed the clinician portal per day for more than two years, while the app was rated as “highly usable”, achieving a mean System Usability Score score of 85.74 (SD 12.37), which translates to an adjective rating of “Excellent”. The participants also gave high scores on “ease of use and learnability” with an average score of 4.33 (SD 0.65) on a scale of 1 to 5.

**Conclusions:**

iREST provides a feasible platform for the implementation of Just in Time Adaptive Intervention in mHealth-based and remote intervention settings. The system was rated highly usable and its cross-platformness made it readily implemented within the heavily segregated smartphone market. The use of wearables to track sleep is promising; yet the accuracy of this technology needs further improvement. Ultimately, iREST demonstrates that mHealth-based Just in Time Adaptive Intervention is not only feasible, but also works effectively.

## Introduction

Insomnia is a prominent sleep problem. Defined as “a difficulty in falling asleep, difficulty staying asleep, and non-restorative sleep,” [1] insomnia can contribute to further symptoms upon waking such as fatigue, impaired concentration, and mood disturbance [2]. Approximately 30% of adults in the United States have at least one of the symptoms of insomnia [3]. Diagnostic rates of insomnia however—based on criteria from the *Diagnostic and Statistical Manual of Mental Disorders, Fourth Edition (DSM-IV)* —range between 5%-20% in the general adult population [4] and 20%-30% in primary care medical settings [5,6].

Since insomnia poses serious mental and physical health hazards, developing more efficacious treatment options is imperative. In general, there are two types of treatment for insomnia: pharmacological and behavioral. Hypnotic agents such as benzodiazepine receptor agonist (BZRA) drugs, are widely available, easy to use, and have rapid and sustained efficacy [7]. BZRA and other pharmacological treatments however, may lead to dependence and substance abuse [8]. Recently, studies have found that behavioral treatments such as Cognitive Behavioral Therapy for Insomnia (CBTI) [9] can be as effective as pharmacological treatments [10]. Furthermore, these behavioral treatments are often preferred by patients [11] and have been shown to have both short-term and long-term efficacy [12,13] with few apparent adverse effects.

Still, despite evidence that sleep disturbances are a modifiable threat to psychological and physical health, the use of evidence-based behavioral sleep treatments remains limited. By far the most limiting factor in making CBTI widely available is the shortage of trained clinicians. Although CBTI typically lasts for only eight sessions, a licensed psychologist trained in behavioral psychology must conduct these sessions [6]. Such restrictions pose an impediment to providing CBTI since there is currently a critical shortage of clinicians trained in evaluating and effectively treating sleep disturbances using behavioral strategies. Furthermore, there is also a geographic barrier to care: trained behavioral sleep clinicians are concentrated mostly in just a few major cities, while the need for them is dispersed throughout the entire country, especially in rural areas.

In response to these challenges, several internet-based solutions have emerged. SHUTi (SHUTi, Charlottesville, VA), as an example, provides CBTI through a web-based application. The results of this intervention are promising [14,15]. The SHUTi platform however requires that the user adhere to taking subjective assessments (eg, sleep diaries and quizzes), reading the modules, and watching provided videos. Moreover, SHUTi works on predetermined “if-then” algorithms, thus limiting the option of tailoring the intervention to a wide variety of individual response-to-treatment patterns, environmental contexts (eg, working schedule, daily routine), and condition severities. The rapid adoption of mobile technologies such as smartphones and wearable devices may help in the development of remote behavioral interventions for insomnia. For example, smartphone-based interventions may outperform web-based approaches such as SHUTi because they allow for continuous, prospective offline use, sensor access and integration, two-way messaging between patients and clinicians, and context awareness. Furthermore, wearable and smartphone-based sensors allow for the objective measurement of sleep and wake patterns instead of relying only on bias-prone, subjective sleep diaries.

Current mobile technologies can further tailor insomnia intervention by dynamically adapting both the assessment and intervention. For example, adaptability can be achieved by allowing the clinician to change the amount or the interval of sleep restriction prescribed to a patient in response to changes in the patient’s sleep pattern and working hours. This sort of adaptability requires personalization of the intervention not only at the beginning of the episode of care, but also frequent iterative adjustments during care. When this adaptive intervention is combined with a smartphone-based approach, the result is the “Just in Time Adaptive Intervention” (JITAI) model [16].

An increasing number of studies have been conducted to assess the effect of JITAI on regulating human health behavior [17-21]. No generalizable application platform however, is yet available for JITAI. Such a platform would include ready-to-use, cross-platform, and reusable components like libraries, communication platforms, sensor integration, database access, and a logical infrastructure. The platform would allow application developers to customize the architecture to support a variety of health behavioral change interventions without having to build the system from scratch.

This work therefore aims to develop a JITAI application platform for an implementation in behavioral sleep interventions. This ostensible application has been called “interactive Resilience Enhancing Sleep Tactics,” or iREST.

## Methods

### Preliminary Works

In the Health and Rehabilitation Informatics Laboratory at University of Pittsburgh, we have developed a JITAI platform—that is, a generalized platform conducive for work on a variety of health-intervention cases such as depression, anxiety disorders, smoking cessation, weight management, chronic condition management, and insomnia [22-25]. From a technological perspective, the difference between implementation across these health-intervention cases occurs mainly in the content of data, information presentation, and data collection methods (wearable devices used); however, the communication infrastructure and the service-oriented architecture remain constant across all cases.

### Study Design

#### Phase 1: Development of iREST System

In this study, we developed the iREST system based on our JITAI platform in accordance with the needs of a behavioral sleep intervention study. In implementing the JITAI platform into the iREST system, we have followed the Iterative and Incremental Development (IID) software development model [26].

Requirement analysis was performed based on traditional behavioral insomnia workflows, clinician inputs, as well as a previous Brief Behavioral Therapy for Insomnia (BBTI) (a shorter, but equally effective version of CBTI), a military study, [27] and a previous implementation of the JITAI platform [24]. This analysis process was focused on determining the “context” and the “contents” of a JITAI delivery system for behavioral sleep intervention. A context is the type of health or behavioral therapy on which JITAI is implemented, for example: child anxiety, depression, insomnia, smoking cessation, and weight management. Contents, on the other hand, comprise such things as assessments, education materials, and guidelines needed to be communicated to achieve the goals of each context.

JITAI’s requirement (Multimedia Appendix 1) for self-administered measurements has necessitated the development of numerous metrics in the iREST system. In iREST, users must fill out an electronic sleep log [28], a weekly assessment regimen, the Patient Health Questionnaire [29], the Asberg Rating Scale for side effects [30], and the User Global Impression of Improvement [31]. Additionally, clinicians must be able to access and complete the Clinician Global Impression [32] on a weekly basis so as to chart the user’s ostensible progress.

Moreover, in using the JITAI platform to develop an effective iREST application, other functional requirements such as reminders and notifications, multimedia education and information delivery, real-time communication, and automatic data collection must be considered. These functional requirements have necessitated an integration of technologies like push-notifications, a secure messaging system, and wearable or Fitbit (Fitbit, San Francisco, CA) interface respectively.

Several of JITAI’s non-functional requirements must also be accounted for and implemented during the development of the iREST app. These non-functional requirements include: privacy and security implementations, cross-platform capability, access and distribution concerns, safeguards for assurance and reliability, and a method for maintaining a separation of concerns. Addressing these non-functional requirements necessitates integrating an encryption technology, offering the app on various digital marketplaces like Google Play (Google, Mountain View, CA) and the Apple App Store (Apple, San Jose, CA), and developing a recovery procedure in the event of service disruption to minimize downtime.

#### Phase 2: Usability Evaluation

A pilot study was conducted to evaluate the usability of the iREST mobile health (mHealth) tool. The purpose of the usability study was to reveal how real patients and clinicians interact with the iREST system, gather their feedback, and improve the system based on the results.

The University’s Institutional Review Board approved the present study. Participant recruitment and screening were conducted by the University of Pittsburgh Military Sleep Tactics and Resilience Research Team. Active Duty Service Members (ADSM) and Veterans between the ages of 18 and 60 were recruited through postcard, flyer, study website, social media and Facebook (San Francisco, CA) and public television. Since the study is a “Bring Your Own Device” (BYOD) study, to be eligible, ADSM and Veterans had to own a smartphone with internet access and be fluent in the use of a smartphone. Other eligibility criteria included:

1. Endorsing significant sleep complaints as determined by a baseline score higher than 5 on the Pittsburgh Sleep Quality Index (PSQI) [33];
2. Having a baseline score greater than 10 on the Insomnia Severity Index (ISI) [34]
3. Having sleep complaints for at least 1 month.

Exclusion criteria included:

1. A history of psychotic disorder or bipolar disorder
2. Suspected or previous diagnosis of sleep apnea narcolepsy or other sleep disorder requiring further evaluation and treatment;
3. Severe or untreated psychiatric disorder associated with marked impairments in functioning
4. Being pregnant or lactating
5. A scheduled/imminent military deployment during the study.

During the first office visit, participants were required to participate in a tutorial on how to use the iREST app. After this first visit, participants could try the app for 7-10 days. Following that period however, participants were required to return to the office to complete a “first impression” usability questionnaire as well as to provide feedback about the app in general. Afterwards, participants were instructed to continue using the app for the next 4-6 weeks of their BBTI. After this 4-to-6–week period, participants again returned to the research office for a postintervention usability assessment using the same assessment tools as those performed during the return visit (first impression). Those usability questionnaires implement in the study were the System Usability Scale (SUS) [35] and a modified version of Telerehabilitation Usability Questionnaire (TUQ) [36]. The SUS is a simple, ten-item scale giving a global view of subjective assessments of usability, while the TUQ—which is currently undergoing validation—measures several usability factors, including usefulness, usability, effectiveness, reliability, and satisfaction. Twenty-one questions were derived from previously validated questionnaires, including the Technology Acceptance Model, Perceived Usefulness and Ease of Use, the Telemedicine Satisfaction Questionnaire, and the Post-Study System Usability Questionnaire/Computer System Usability Questionnaire. In addition to formative usability questionnaires, participants were also asked to provide quantitative feedback or comments about the use of the iREST app. Measuring the system usability twice, before and after intervention, allowed for observation of whether habituation affects participant perception of the system’s ease-of-use. We hypothesized that habituation would not significantly affect usability in a negative way. Therefore, a paired Student t-test was performed to compare the SUS and the TUQ scores, preintervention and postintervention.

Adherence was calculated as half of the total participant logs (half, because there are two logs each day: wake log and sleep log) over the total number of days that participants used the app in the study. The completion time for each log was calculated by measuring the time lapse between the moment in which participants began accessing the sleep/wake log screen and the moment in which they hit the save button (for example, completed the logs). The calculation is performed automatically by the iREST system. In addition, the overall usage was estimated by calculating the number of unique devices with an iREST app accessing the iREST server per day.

#### Phase 3: Wearable Sensor Integration and Evaluation

In addition to the usability evaluation, we also evaluated the feasibility of further improving participant experience by using wearable sensors, which can potentially remove the burden of entering sleep diary data manually. To explore this potential, seven Fitbit Charge wristbands were randomly assigned to participants. Participants assigned with Fitbit bands were required to wear the band to measure their sleep patterns in addition to filling out the in-app sleep diary. After the BBTI intervention, sleep diary and Fitbit-reported sleep data were compared. This was meant to measure the degree of agreement between sleep parameters reported subjectively by the participants and measured by Fitbit devices: a high degree of agreement would indicate a higher potential for Fitbit to replace the need of manual sleep diary entries in our future BBTI interventions.

IBM (Armonk, NY) SPSS Statistics software version 24.0 was used for data analysis for Fitbit vs sleep diary comparison, while GraphPad (La Jolla, CA) Prism version 7 was used to build Bland-Altman plots. Sleep diary and Fitbit-reported sleep data were compared. First, intraclass correlation coefficients (ICC_2,1_) were used to examine agreement between sleep parameters taken from the Fitbit and sleep diary data. An ICC ≥0.75 was considered excellent, 0.60–0.74 good, 0.40–0.59 fair and<0.40 poor [37]. A Bland-Altman plot [38] was used to visualize any systematic difference between values reported by the two measurements.

## Results

### Development Results

**Figure 1:**
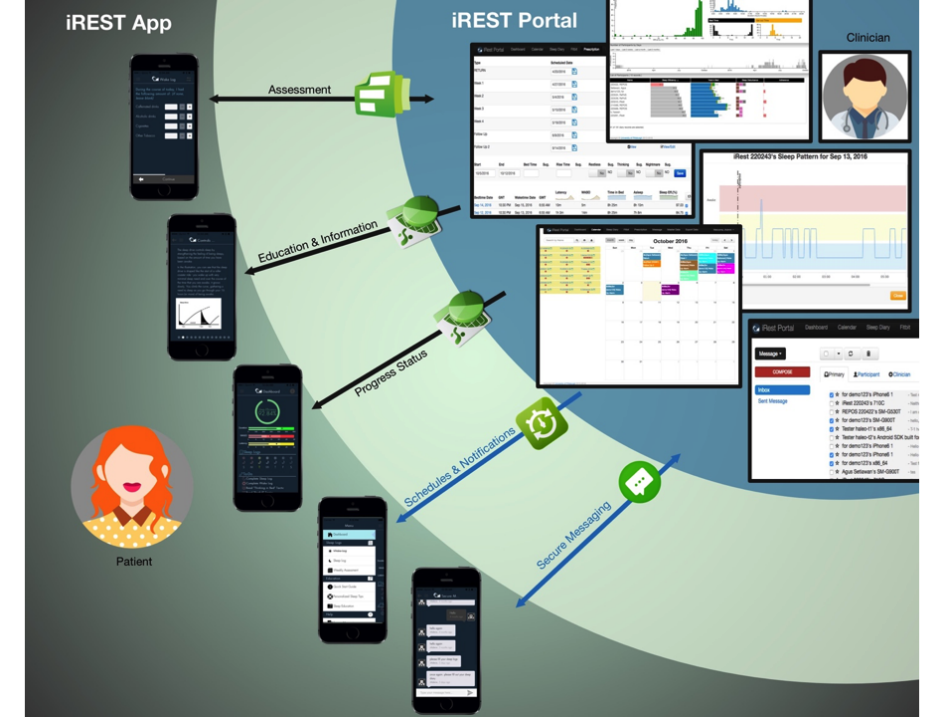
A model representing the iREST app and clinician-portal’s two-way interactions which include: assessment, education/information delivery, progress reporting, scheduling, notification delivery, and secure messaging.

Currently, the iREST system (Figure 1) consists of a cross-platform smartphone app, a clinician portal, and a secure 2-way communication platform that connects the app and the portal.

### Mobile Application (App) Features

**Figure 2:**
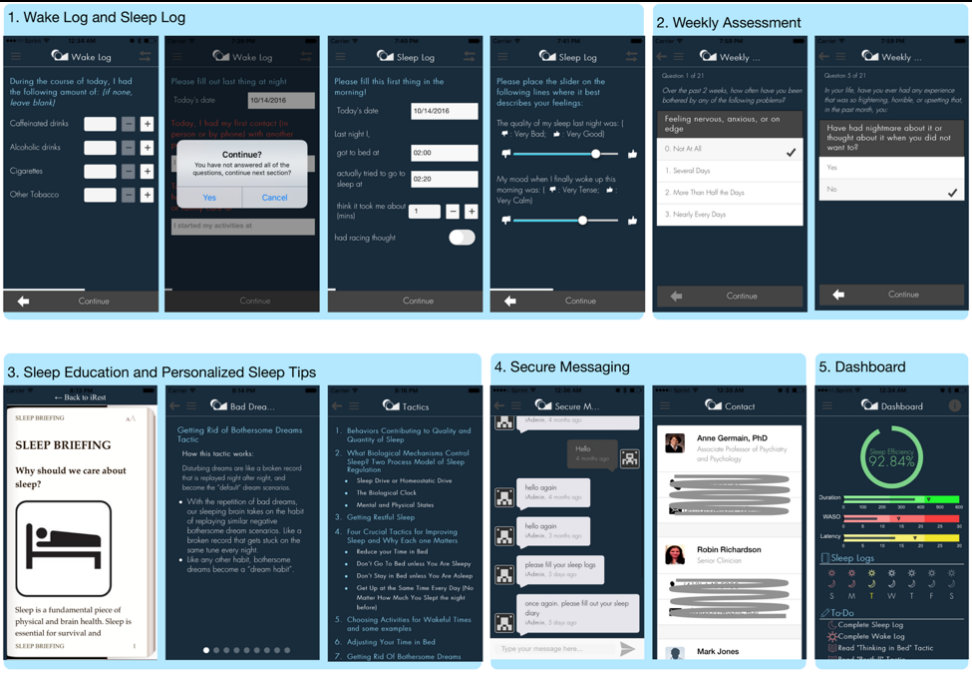
Screenshots of the various features implemented in the iREST app. 1) Wake activity and sleep pattern recording part of the iREST app, participants need to enter each section once a day; 2) Weekly assessment was scheduled each week to measure participants’ clinical progress; 3) Snapshots of various sleep education and personalized tactics that the app provides based on each participant’s condition; 4) Secure massaging feature that allows participants and clinicians to exchange information securely, eg, instead of using short message service/text. 5) The iREST app’s dashboard, showing an at-a-glance view of individual participant current status and progress.

The iREST mobile app (Figure 2) is used by the patient to record sleep data, present feedback and related education materials, and provide cues and notifications. Following are the app’s main features:

#### Wake Log and Sleep Log

The wake log and sleep log of the iREST app is an electronic adaptation of the Pittsburgh Sleep Diary. The wake log records users’ daytime activities that may impact healthy sleep practices. Such effecters include caffeine and alcohol consumption, number and duration of daytime naps, and exercise events. Users are intended to complete the wake log right before going to bed. Conversely, the sleep log tracks users’ sleep parameters—including sleep latency, number and duration of wake-up after sleep onset episodes, bedtime, and wake time—dreams or nightmares, and perceived sleep quality. Users are required to fill out the sleep log immediately upon awakening to reduce recall bias.

For both sleep and wake logs, the app records time-stamps at the commencement and completion of each entry. Furthermore, the logs implement “validation checking”, a function meant to ensure the thorough completion of app tasks. For example, while the user completes the logs, validation checking immediately alert the user if they have made any mistakes or missed any fields. Moreover, validation checking uses the previous entry as a default value for each new entry to reduce time and user burden in filling out the logs.

#### Weekly Assessment

The weekly assessment is a regimen of assessments administered to users on a weekly basis. These assessments consist of the Generalized Anxiety Disorder scale 2-items [39], the Patient Health Questionnaire-2 [29], the Patient-Rated Global Impression of Improvement [31], and a modified Asberg Rating Scale [30] side effect questionnaire that measure both the users’ weekly progress and any potential side effects from treatment. This assessment appears to the user only when the clinician schedules it.

#### Sleep Education and Personalized Sleep Tips

Sleep education contains information about sleep, insomnia, brain sleep-mechanisms, and healthy sleep practices. These educational materials are always available on the iREST app. Additionally, the personalized sleep tips offer specific information on how to address or overcome certain behaviors, cognitions, or events (like nightmare episodes) that may be perceived as barriers to healthy sleep. Clinicians can prescribe sleep tips based on reports from users’ sleep/wake logs. For example, if a user reports having a nightmare, the clinician can prescribe tips aimed at “getting rid of bothersome dreams” directly to the user’s iREST app.

#### Secure Messaging

Secure messaging allows real-time message exchange between clinicians and users while maintaining high privacy and security—two factors that are often lacking on regular text messaging and short message services. Multiple security measures are implemented in the secure massaging feature, including a strong protocol for communication between the iREST app and server using Transport Layer Security, a secure encryption key exchange, an encoding and enciphering of messages, and achieving an encrypted database behind a firewall.

The secure messaging feature allows users and clinicians to exchange information that may not be readily available through the app’s other functionalities. For example, through secure messaging—after reading the personalized sleep tips prescribed by the clinician—a user may request additional information on specific sleep problems. The clinician can then reply with links to additional resources.

#### App Dashboard

The iREST app’s dashboard provides “at-a-glance” views of key performance indicators on individual treatment progress. In sleep interventions, these indicators can be sleep parameters such as sleep efficiency, sleep latency, wake-up-after-sleep-onset, and total sleep time. The dashboard also contains indicators of logs and assessment completion. In addition, the dashboard provides visual notification for new messages and new tips that are received from the clinician portal.

### Clinician Portal Features

**Figure 3:**
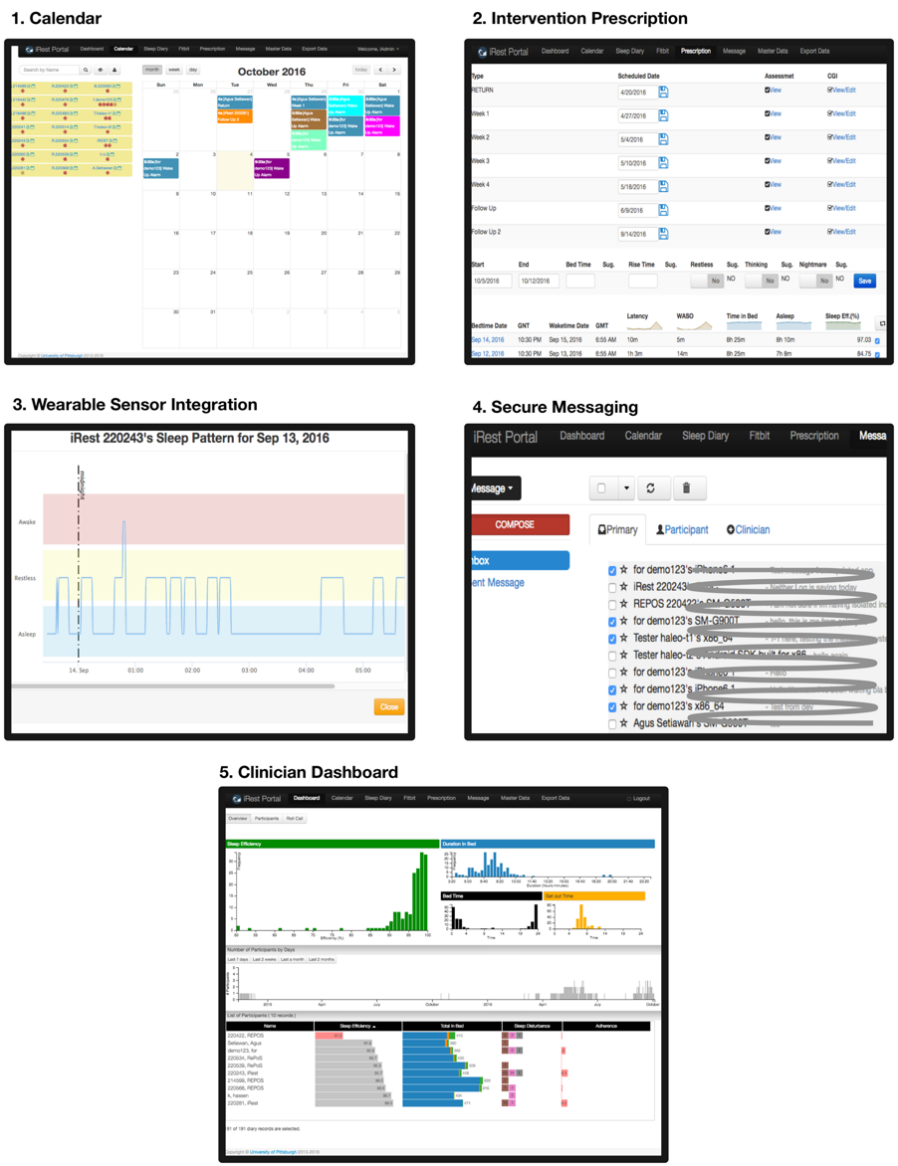
Screenshots from the iREST clinician portal. 1) The portal’s calendar view which simplify the way clinicians manage participants’ scheduling; 2) The prescription window where clinicians can view individual participant’s status, view treatment suggestions calculated by the system, and prescribe appropriate intervention; 3) An example of a participant’s sleep pattern retrieved from Fitbit; 4) Secure messaging on the clinician side; 5) The clinician’s dashboard where a clinician can view the whole status of participants under their care, see the clinical indicators/signs of progress and prioritize treatments.

Like the iREST mobile app, the clinician portal (Figure 3) is an implementation of the portal portion of the JITAI application architecture, based on the requirements needed by the clinicians to fully support the intervention (as also described in Multimedia Appendix 1). Below are the main features available in the iREST clinician portal:

#### Clinician Dashboard

The iREST portal’s dashboard provides data visualization of users’ progress in the intervention, the intervention status as whole, and general views of the mHealth utilization. It allows clinicians to make priorities on resource allocation based on the severity of users’ conditions. For example, users who frequently express sleep problems will have more clinician time than users whose interventions are going well.

#### Calendar and Scheduling

The calendar view allows clinicians to quickly assess the status of scheduled intervention components such as prescribed wake time and assessment schedules. This page also provides users with a mobile device status (active, idle, inactive) and shortcuts for creating new schedules for sending secure messages to users.

#### Intervention Prescription

Intervention prescription is the main feature for managing and prescribing intervention components. It provides users’ daily sleep logs and weekly progress summaries. Based on these summaries, the portal suggests appropriate sleep prescriptions, and the clinicians then make judgments on which course of action to take, or which intervention components to prescribe to the users’ mobile app.

#### Wearable Sensor Integration

With the current clinician portal, only integration with Fitbit is supported. The integration functionality provides interfaces to perform sleep data imports from the Fitbit server.

### Participants

**Figure 4:**
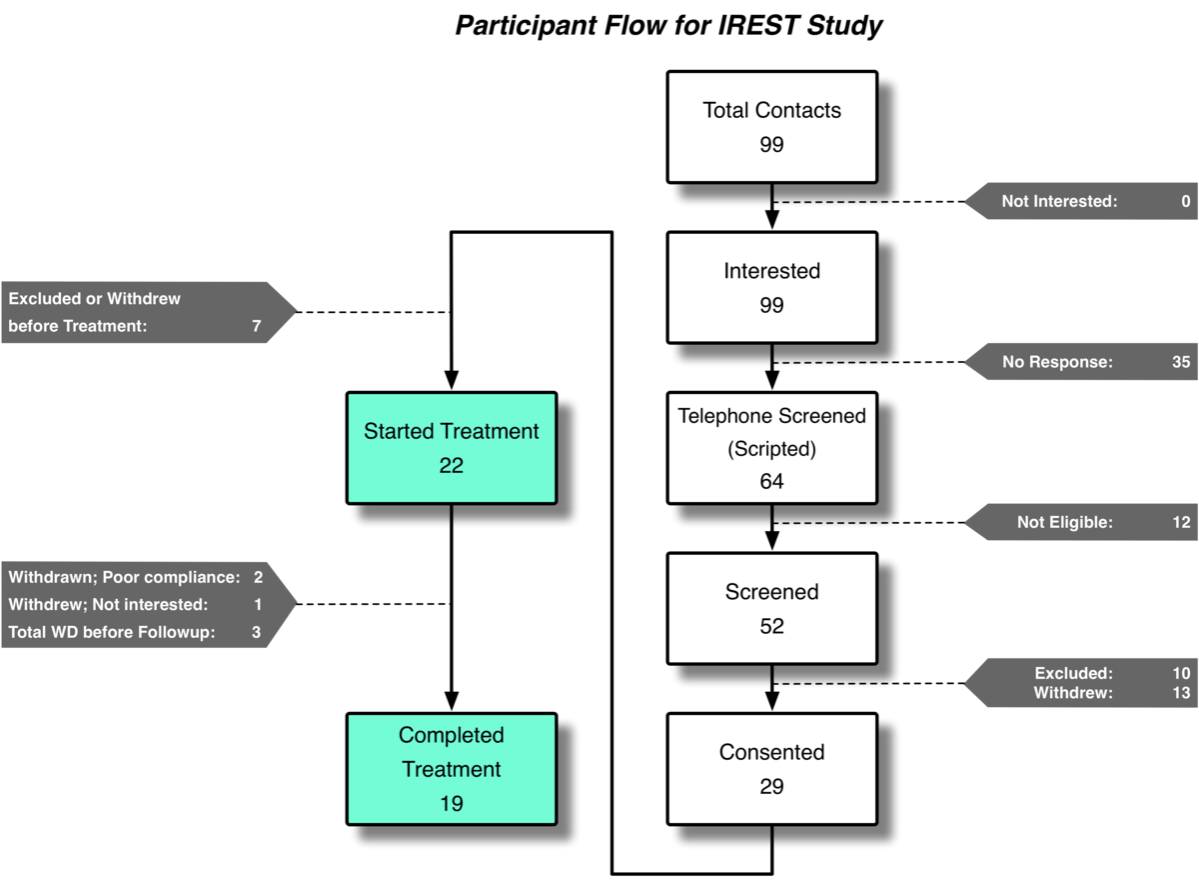
iREST Usability Study's Participant Flow. WD: withdrawn.

As shown in Figure 4, a total of 99 individuals contacted the research program to inquire about the study, all expressing interest in participating. During the scripted telephone screening, 35 (36%) individuals did not respond after several attempts to contact them. Twelve individuals (19%) were found not eligible after telephone screening. ADSM and Veterans who passed the telephone screening attended the in-office diagnostic evaluation; ten individuals (19%) were excluded in this phase. Twenty-nine ADSM and Veterans provided written informed consent; however, seven of them (24%) withdrew from the study before the intervention. Out of 22 who started the intervention, nineteen (19) participants (86%) completed posttreatment and follow-up assessment. Six (32%) participants used an iPhone or iOS device, and the other 13 (68%) used an Android device.

Descriptive statistics were performed to describe the demographic characteristics of the study participants using frequencies for categorical variables, means, and standard deviations for continuously measured demographic variables. Demographic information obtained at baseline is provided in Table 1.

### Usage Characteristics

**Figure 5:**
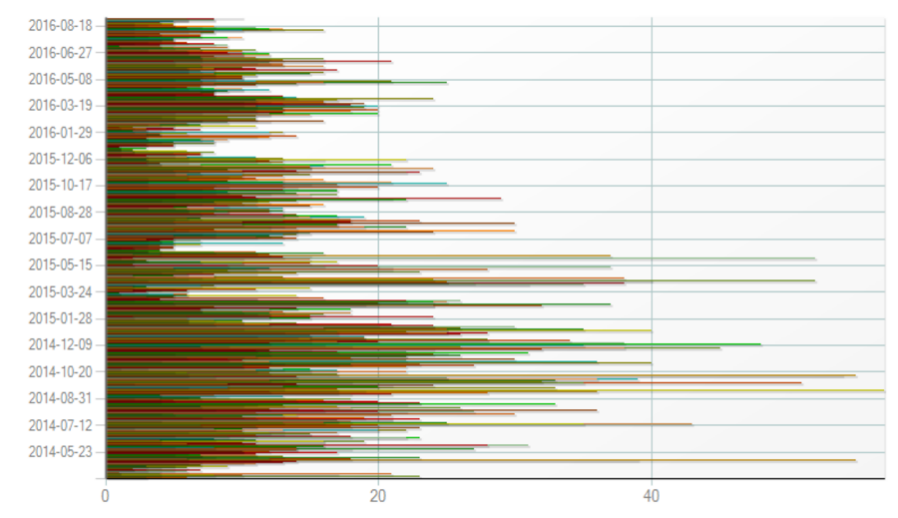
Statistical representation of daily unique device access to the iREST portal. Each line represents number of unique participants accessing the portal for each day.

One way to describe the overall usage of the system is by calculating the number of unique devices accessing the iREST portal per day. As seen in Figure 5, on average, there were at least 12 (mean 12.23, SD 8.96) unique devices accessing the portal daily for more than two years following the iREST study commencement. On the app side, according to the Apple App Store and Google Play statistics from September 2016, the app was downloaded and installed 247 times (182 on Android [Google, Mountain View, CA] and 67 iOS). The number of downloads was significantly higher than the number of participants in the study (in total only 29 participants downloads), which may indicate that there was high demand for a sleep or insomnia app on the market. Currently, the app is active on 53 devices (47 Android and 6 iOS). In addition to the current study, the iREST mHealth system was also used to support at least two other sleep research studies.

**Table 1:** Demographic and clinical baseline.

Variable	Value
Male, n (%)	18 (82)
Caucasian, n (%)	17 (77)
Age, mean (SD)	38.7 (9.7)
Army, n (%)	14 (64)
Pittsburgh Sleep Quality Index, mean (SD)	11.9 (3.9)
Insomnia Severity Index, mean (SD)	17.4 (4.0)
Epworth Sleepiness Scale, mean (SD)	7.4 (4.6)

During the study, our online server experienced an unplanned outage resulting in no data collection over a three-day period for two participants. This unexpected problem was subsequently fixed by making the system capable of handling server and connection outages. Even with this outage, on average participants completed 91.11% of the required twice-a-day sleep diary entries, only failing to fill out less than 3 days’ worth of sleep diaries throughout the course of the study. This adherence percentage is significantly higher than the average technology-mediated insomnia treatment adherence of 52% [40]. It took an average of less than two minutes (108.53 seconds, SD 26.19) to complete each assessment.

### Usability Results

In the postintervention follow-up visit, 17 out of 19 participants finished the poststudy questionnaires (the SUS and the TUQ). The sample size is considered appropriate according to the Problem Discovery Rate Model, which is widely used to serve in formative usability evaluations [41,42]. According to the model, 85% of usability problems were revealed using five participants, and almost 100% of problems using 14 participants [43]. The participants rated the app as highly usable with a mean SUS score of 85.74 (SD 12.37), which translates to adjective ratings of *“Excellent”* [44]. On the TUQ, participants were satisfied with the iREST app and would consider using it in the future (average score of 4.31 out of 5, SD 0.63). They also gave high scores on “ease of use and learnability” with an average score of 4.33 (SD 0.65). In assessing room for improvement, the sections for “interface quality” and “reliability” received slightly lower scores, although still above average, with a mean score of 4.05 (SD 0.85) and 3.88 (SD 0.70), respectively. Server outage may have contributed to lower scores on reliability, while the lower interface quality score shows the need for more meaningful data visualization and better overall user-interface design.

When compared with pretreatment scores, both SUS and TUQ posttreatment scores were higher. The results show that participants continued to rate the iREST app as highly usable even as they became more familiar with the system; in other words, rather than fostering *contempt*, in this case, familiarity can be said to *breed contentment.* Furthermore, the improvement in the interface quality score on the TUQ was statistically significant (Table 2), and a noticeable score increase was observed on Reliability (mean increase of 0.45). Continued user-centered improvements (eg, incorporating users’ feedback and addressing user interface, UI, interaction problems) in user interface and system reliability most likely contributed to the noticeable increase in TUQ scores for those two areas.

On the qualitative usability assessment, participants provide individual comments and feedback about the app. Responses were generally categorized into five types:

1. General comments about the app
2. Comments about the graphical user interface and navigation
3. Comments about the sleep logging process
4. Comments about sleep education features
5. Questions and problem reporting.

Participants expressed liking the application generally with reported comments such as: *“[I] like the front page a lot, [I] find it useful and attractive”*; *“[The app is] very easy to navigate, [I] found that the data uploaded quickly”*; *“[I] like the morning reminder to fill out wake time diary.”* Participants also pointed out issues and made suggestions, such as: *“[The app’s format for time input was tedious, [this] needs improvement”*; *“[I was] frustrated by [the] text overlap [that occurs] when [the] device is held horizontally”*; *“[Developers should] have the SE% graphic replaced by something, eg, tracking how many logs were entered on time.”* Multimedia Appendix 2 contains a portion of the reported feedback and comments. This feedback was used to iteratively improve the iREST app and as input for future developments.

Additionally, qualitative analysis of participants’ feedbacks on iREST app identified several potential improvements and additional features suggestions. Most of the critical suggestions have already been addressed and incorporated into the app during the development iterations. Some suggested improvements however remain to be addressed in future developments. These include:

1. An informative and concise, but customizable data visualization on the app’s dashboard
2. A smart data input, in which the app learns from previously-entered data about each participant’s usual sleep habits to reduce participant burden
3. Include general UI components
4. Implementing more reliable notifications and reminders
5. Streamlining the Fitbit integration

**Table 2:** A paired *t* test comparison of Usability Questionnaires Scores (System Usability Scale and Telerehabilitation Usability Questionnaire) between pretreatment and posttreatment. SUS: System Usability Scale; TUQ: Telerehabilitation Usability Questionnaire.

Variable		Before treatment (SD)	After treatment (SD)	Pretreatment to postmean score change		
				Estimates (SE)	*t* statistics (*df*)	*P* value
SUS		78.04 (13.66)	85.74 (12.37)	6.61 (3.65)	1.81 (13)	.09
**TUQ**						
	Ease of use	4.15 (0.55)	4.33 (0.65)	0.14 (0.18)	0.76 (10)	.47
	Interface Quality	3.65 (0.65)	4.05 (0.85)	0.55 (0.17)	3.25 (10)	.009^a^
	Interaction Quality	3.77 (0.76)	3.95 (0.78)	0.20 (0.14)	1.11 (10)	.29
	Reliability	3.58 (0.53)	3.88 (0.70)	0.45 (0.22)	2.09 (10)	.06
	Overall Satisfaction	3.96 (0.80)	4.31 (0.63)	0.36 (0.22)	1.62 (10)	.14

^a^Statistically significant.

**Table 3:** Fitbit versus iREST Sleep Diary.

Variables	Fitbit, mean (SD)	iREST Diary, mean (SD)	Mean difference (SE)	*P* value	ICC_2,1_ (range)
Sleep onset latency (minutes)	0.38 (0.38)	18.60 (1.63)	18.23 (1.66)	<.001	0.15 (-0.123-0.153)
Wakefulness after sleep onset (minutes)	5.64 (0.58)	20.19 (1.97)	14.54 (1.86)	<.001	0.174 (0.038-0.305)
Total in bed (minutes)	422.9 (6.48)	430.9 (6.00)	8.08 (4.80)	.09	0.705 (0.628-0.768)
Total sleep time (minutes)	416 (6.39)	392.14 (6.82)	–24.69 (4.79)	<.001	0.737 (0.667-0.794)
Sleep efficiency (%)	98.59 (0.16)	90.53 (0.74)	-8.06 (0.70)	<.001	0.144 (0.006-0.276)
Good night/fall asleep time	11:42:12PM (5.55)	11:27:06PM (5.35)	–15.10 (3.95)	<.001	0.738 (0.668-0.795)
Good morning/awake time	06:45:00AM (6.87)	06:38:00AM (6.25)	–7.03 (4.39)	.11	0.777 (0.715-0.826)

### Fitbit Integration

Seven participants were assigned with Fitbit Charge throughout the course of the usability study. In total, 202 paired (Fitbit vs sleep diary) nights were acquired. We utilized the automatic sleep detection feature available on the Charge model, in which the wristband automatically detects when the wearer falls asleep and wakes up without manual input (eg, pressing a button). This feature, although convenient for participants, greatly underestimates latency to onset of the first sleep epoch (sleep onset latency, SOL). As a result, Fitbit only reported one instance of SOL (SOL>0 minute) out of 202-recorded nights. Due to limitations in statistical analysis packages, variables representing clock time, such as Good Night Time (GNT) and Good Morning Time (GMT) are translated into minutes-distant-from-midnight (12:00 AM). For example, 11:00 PM on GNT was translated into “–60” (60 minutes before midnight), and 5:15 AM in GMT was translated into “315” (315 minutes after midnight).

As shown in Table 3, significant statistical differences were found for the following variables recorded between Fitbit and sleep diaries: latency (SOL), wakefulness after sleep onset (WASO), total sleep time (TST), GNT, and sleep efficiency. With diaries recording longer means of latency, longer means of WASO, shorter means of TST, earlier GNTs and smaller average sleep efficiencies. No significant differences however were found on total in bed (TIB; longer in sleep diary) and GMT (earlier in sleep diary). Furthermore, good intraclass correlations were observed for TST (ICC_2,1_=0.737, *P*<.001), GNT (ICC_2,1_=0.738, *P*<.001), and TIB (ICC_2,1_=0.705, *P*<.001). There was excellent agreement on GMT between Fitbit and sleep diary entries, with ICC_2,1_=0.777, *P*<.001.

**Figure 6:**
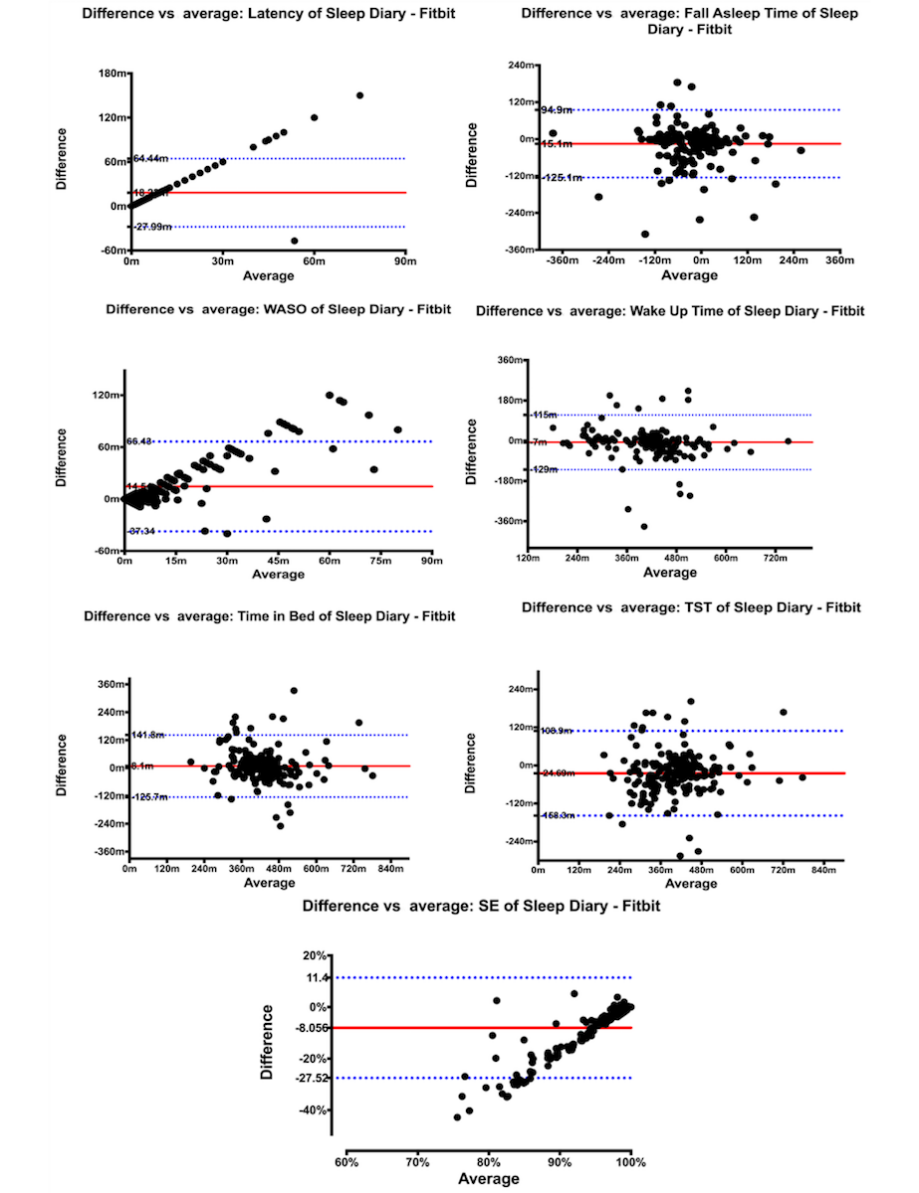
Bland-Altman plots of Sleep Diary vs. Fitbit. SE: sleep efficiency; TST: total sleep time; WASO: wakefulness after sleep onset.

Bland and Altman difference plots (Figure 6), for these sleep parameters showed no statistically significant agreement between Fitbit and sleep diaries. As demonstrated in ICC analysis, the plots also show a higher level of disagreement between Fitbit and diaries for SOL, WASO and sleep efficiency. Moreover, proportional bias was observed for these three variables, and the disagreement between measurement modalities increased as the average value of SOL and WASO increased, and as the average value of sleep efficiency decreased from 100. It was observed that when an individual’s day-to-day sleep pattern variability increased, the level of agreement between Fitbit and sleep diaries decreased for that individual when compared with the group mean. For example, a participant whose WASO changed significantly from night to night (eg, from 0 on the first day, to 45 on the second day, and back to 15 on the third) is likely to have worse agreement on the Fitbit vs sleep diary WASO when compared with the rest of the sample.

## Discussion

### Principal Findings

The JITAI application architecture used in iREST gives potential leverage for intervention-scientists in implementing mobile app solutions for other JITAI based projects. The architecture provides a wide variety of functionalities, design patterns, and guidelines that are readily implemented in various JITAI mobile app solutions. Also, the JITAI application architecture is cross-platform and therefore allows rapid deployment to various mutually incompatible mobile operating systems and opens the possibility for a BYOD approach, a feature that greatly increases the scalability of, and access to, interventions.

The usability evaluation of iREST showed that the app is highly usable and supports high adherence to treatment regimens. In addition, the evaluation allowed the detection of potential improvement of the iREST system based on participants’ feedback and comments about the system during the usability study. These improvements have been incorporated in subsequent development iterations of iREST system.

The usability results demonstrated that not only is the IREST app applicable to implementing BBTI in a military population but is also usable and well received. Overall, participants were satisfied with the iREST application, finding it easy to use. All nineteen participants used the app daily to record their sleep with very few missed entries (on average less than 3) over the course of the 4-to-6-week intervention. The iREST app’s sleep and wake logs were optimized for touch-based input, which reduced fill-out time and participants’ burden.

Consistent with previous studies conducted in the use of movement-based sensors (eg, Fitbit, actigraphy) for measuring sleep [45–47], we found poor agreement between Fitbit and participants’ reported sleep diaries, although clinically, the Fitbit data may be sufficiently used as a consideration for BBTI sleep prescriptions (eg, mean differences for sleep parameters between Fitbit and sleep diary are below the clinically significant threshold of 30 minutes). Improvements nevertheless need to be made to the architecture to increase the sensitivity of Fitbit’s measuring of sleep parameters. As a possible solution for the next iteration of the iREST system, we plan to use a hybrid approach between the two modalities, to incorporate a machine learning algorithm and to allow participants to modify Fitbit reported data. Each modification will then feed into a machine-learning algorithm, so that the longer an individual uses the system, the less modification that will be needed to provide more accurate data.

### Limitations

The present study was highly focused on patients’ improvements and experiences in using the iREST system. The clinicians’ perspectives however, are equally important. As mentioned before, the highly manual nature of BBTI supported by the easy-to-use iREST system is likely to facilitate the delivery of this treatment by mid-level (non-doctoral) clinicians; however, the present study has not provided a sufficient level of heterogeneity in a clinician sample to determine whether a comparable magnitude of improvement would also be observed with less experienced therapists. In the current study, two clinicians with extensive experience in behavioral sleep treatments administered the intervention.

Moreover, although this paper included a usability study and utilization analysis on the iREST patient app, no usability data from the clinician portal was explicitly reported. Clinician usability is something that might be investigated in future studies.

Currently, the iREST wearable integration only supports Fitbit devices. Furthermore, the iREST app does not support background services (ie, running in the background and without user intervention), which would allow the app to keep running after the smartphone screen is locked and the app minimized. This feature is important for real-time communication such as initiation of video or audio calls. Future development to implement this feature has been planned.

### Conclusion

The iREST system provides a feasible platform for implementation of JITAI in mHealth-based and remote intervention settings. The use of Fitbit as an objective measure for ambulatory sleep pattern assessment showed promising results, yet further improvement is needed.

Ultimately, iREST demonstrates that mHealth-based JITAI model works effectively while achieving an excellent usability rating. Although the current implementation was only aimed at insomnia treatment, iREST has the potential to be deployed towards other behavioral health interventions. Furthermore, the promising results in the current pilot study, open the pathway for larger study on clinical feasibility of iREST intervention.

## Appendix

Multimedia Appendix 1 iREST System Requirements.

Multimedia Appendix 2 Participant Feedbacks and Comments on iREST App.

## Abbreviations

ADSM: Active Duty Service Members
BYOD: Bring Your Own Device
BBTI: Brief Behavioral Therapy for Insomnia
BZRA: benzodiazepine receptor agonist
CBTI: Cognitive Behavioral Therapy for Insomnia
DSM IV: Diagnostic and Statistical Manual of Mental Disorders, Fourth Edition
GNT: Good Night Time
GMT: Good Morning Time
ICC: intraclass correlation
JITAI: Just in Time Adaptive Intervention
mHealth: mobile health
PSQI: Pittsburgh Sleep Quality Index
RCT: randomized controlled trial
SE: sleep efficiency
SOL: sleep onset latency
SUS: System Usability Scale
TIB: total in bed
TST: total sleep time
TUQ: Telerehabilitation Usability Questionnaire
UI: user interface
WASO: wakefulness after sleep onset
