# Supplementary material for: Development of a Just-in-Time Adaptive mHealth Intervention for Insomnia: Usability Study
Source: JMIR Hum Factors. 2018 May 17;5(2):e21. doi: 10.2196/humanfactors.8905 (PMC5981058; doi:10.2196/humanfactors.8905)
Supplement: Multimedia Appendix 1 [file humanfactors_v5i2e21_app1.pdf]

Multimedia Appendix 1. iREST System Requirements

Table 1. The functional requirement analysis for iREST app.

**Functional Requirements**	**Description**
Separation between roles	Users, clinicians, administrative staff must be given different access rights to the system
Self-administered measurements	For the intervention, users need to be able to complete a number of metrics. These include:<br>1. An electronic sleep log [28]<br>2. A weekly assessment regimen which contains the Generalized Anxiety Disorder scale 2-items (GAD-2) [39]<br>3. The Patient Health Questionnaire-2 (PHQ-2) [29]<br>4. A modified Asberg Rating Scale for Side Effects [30]<br>5. The patient-rated Global Impression of Improvement (PGI-I) [31]<br>Clinicians must also fill out the Clinical Global Impression scale (CGI) [32] weekly to report their impressions of the users' clinical improvement.
Reminders and notifications	The system should allow clinicians to send reminders to the users' mobile app, reminding them to fill out relevant assessments/logs, as well as adhering to the sleep restriction schedule, completing prescribed reading of condition-related information/education, etc.<br><br>The mobile application should show notifications when the user has received a new message from his or her clinician, new assessments are made available, assessments/logs are submitted successfully, and when a loss of Internet connection occurs.<br><br>Clinicians, on the other hand, should be notified when there are new messages from users, or regarding adverse effect endorsements.

Multimedia education and information delivery	The system should allow clinicians to send condition-specific education and information materials to users' mobile apps. In addition, the mobile app should contain general information about insomnia, sleep, and a quick guide on how to use the education module.
Real-time communication	The system should provide a live-chat capability that allows clinicians and users to exchange messages in real-time.
Automatic data collection	The iREST system should use a Fitbit wristband to collect the users' sleep patterns. Fitbit is preferred due to an open API, relatively affordable price and its leading the market of commercial wearables compared to other vendors.

Table 2. The non-functional requirement analysis for the iREST app.

**Non-Functional Requirements**	**Description**
Privacy and Security	The system should provide encryption throughout the data exchange and managements processes. The system should not store any identifiable data on users' smartphones.
Cross-platform	The intervention should use a bring your own device (BYOD) approach; therefore, the system needs to be cross-platform.
Access and Distribution	The mobile app should be available to users at Google Play and the Apple App Store.
Availability and Reliability	The system aims to be available throughout the intervention period. A recovery procedure should be put into place in the event of service disruption in order to minimize down time.
Separation of concerns	The system should be designed on the separation of concern concepts.
