# Supplementary material for: Development of a Just-in-Time Adaptive mHealth Intervention for Insomnia: Usability Study
Source: JMIR Hum Factors. 2018 May 17;5(2):e21. doi: 10.2196/humanfactors.8905 (PMC5981058; doi:10.2196/humanfactors.8905)
Supplement: Multimedia Appendix 2 [file humanfactors_v5i2e21_app2.pdf]

Multimedia Appendix 2. Participant Feedbacks and Comments on iREST App.

APPCOMMENTS	DATAENTRY	FEATURES	GENERAL
	BUTTONS INSTEAD OF SLIDING SCALES FOR CERTAIN QUESTIONS. APP CYCLES TO NEXT ENTRY WHEN "SUBMIT" IS PRESSED	FIELDS TO INPUT SPEC	
SOMEWHAT FRUSTRATED BY APP SENDING HIM "GIBBERISH" SLEEP PRESCRIPTIONS DAILY IN FLUCTUATING GMT AND GNT	COULD NOT GO BACK AND FIX THE INCORRECT TIME ENTERED FOR 'TIME 1 ACTUALLY TRIED TO GO TO SLEEP'	SLEEPTACTICS PAGES VISIBLE SOME DAYS, GONE OTHER DAYS	SERVER FAILURE OVER WEEKEND?
ALL OF THE "SLEEP BRIEFING INFO" IS ONLY VISIBLE INTERMITTENTLY. "SLEEP SUGGESTIONS" PAGE GONE. CONTINUES TO RECEIVE DIFFERENT SLEEP RX DAILY (IN FORM OF ALARM/ALERTS) BUT NOT IN FORM OF MESSAGES-GETS THESE AS ALERTS	SLIDE BARS DEFECTIVE ON IOS PLATFORM. VERY DIFFICULT TO 'SLIDE' BECAUSE OF IPHONE 'HIGHLIGHT' FUNCTION	CONTINUES TO GET POP-UP NOTIFICATION FOR GMT AT ODD TIMES (I.E. 1150 TODAY), INTERMITTENT	
1. FINDING IT PLUS EXPERIENCE TO USE APP TO TRACK SLEEP 2. LIKES THE MORNING REMINDER TO FILL OUT WAKETIME DIARY -> IS THIS THE MORNING ALARM? 3. RECEIVED PRESCRITION BY APP, BUT IGNORED IT	FINDS THAT THE UP/DOWN ARROWS ON ANDROID ONE TIME-CONSUMING FOR SETTING TIME	ADD REMINDER(OPTIONS) FOR BEDTIME DIARY COMPLETION	
SLEEP RX GAVE SAME GMT/GNT DAILY, BUT CAN'T RESPOND RAPIDLY TO CHANGE IN SCHEDULE	11/28/2014-TRIED TO SUBMIT WAKETIME DIARY AND DID NOT GET "SAVED" MSG.	ALARM-GOES OFF REPEATEDLY EVEN AFTER HE HAS ENTERED HIS WAKETIME DIARY	
GOT NEW PHONE, APP MOVED OVER BY PHONE DEALER, HE WAS 'UNCONFIRMED' ON NEW PHONE, BUT COULD STILL ENTER DATA ON OLD PHONE (EXCEPT FOR DAY IMMEDIATELY AFTER PHONE UPGRADE)			
	LIKES ADDED "TIME WHEEL" FUNCTION FOR ANDROID	ALARM GOING OFF ONLY 1X NOW FOR GMT	
WOULD LIKE TO HAVE APP INCLUDE SOME GRAPHICS-FINDS THE DOWNLOADED ACTIGRAPHY DATA TO BE MOST COMPELLING ARGUMENT FOR MAINTAINING CONSISTENT SCHEDULE. WOULD LIKE TO SEE GRAPHICAL REPRESENTTION OF HIS OWN SLEEP DATA ON APP		WOULD PREFER A MORE USEFUL/CONSISTENT "WAKE UP" REMINDER TO ENTER DATA - OCCASIONALLY FOUIND HIMSELF FORGETTING	STOPPED EXPERIENCING ALARM GOING OFF AT NIGHT. FEELS THAT USING THE APP EVERY DAY HAS CREATED SENSE OF ACCOUNTABILITY

	OK	"FIRST CONTACT" INFO SHOULD BE COMPLETED IN MORNING. WOULD BE USEFUL TO ENTER DATA AS IT HAPPENS	
SHOW TIMES OF SUNRISE AND SUNSET ON AP?	FINE	WORKS WELL	CONTACT INFO FOR CLINICIAN
POSSIBLY INTERGRATING A CALENDER OR ALARM CLOCK OPTION. IE CALENDER TO QUICKLY GET TO A SPECIFIC DATE ON SLEEP DIARY AND ALARM CLOCK QUICK FOR RESETTING ALARM			
I HAD AN ANDROID PHONE AT FIRST, THAT ONE BROKE, AND I SWITCHED TO THE IPHONE. TESTFLIGHT WORKED FOR A WHILE, BUT AFTER IT UPDATED IT STOPPED. I COULDN'T GET INTO ANYTHING & IT KEPT GLITCHING			
	VERY EASY TO NAVIGATE, FOUND THAT THE DATA UPLOADED QUICKLY, NO DELAY WITH THE UPLOAD OF DATA	HAVE THE SE% + GRAPHIC REPLACED BY SOMETHING TRACKING HOW MANY LOGS WERE ENTERED ON TIME, ETC.	LIKES THE FRONT PAGE A LOT, FINDS IT USEFUL AND ATTRACTIVE
SERVER POWERED OFF OVER WEEKEND. PARTICIPANTS WERE UNABLE TO ENTER DATA OVER THAT PERIOD OF TIME + IT WAS NOT CACHED ON THEIR DEVICES-LOST DATA. ALSO, AFTER SERVER PROBLEMS, THE SLEEP BRIEFING MATERIALS UPLOADED WITH MISSING OR REPETITIVE SECTIONS			
	WOULD LIKE TO BE ABLE TO SEE	LINE MAY OR MAY NOT CROSS OUT ITEMS ON TO DO LIST	
	EASY, QUICK-NO SUGGESTIONS		
	DROP-DOWN MENUS CONTINUE TO DEFAULT TO A DIFFERENT CHOICE THEN THE ONE THAT PARTICIPANT CHOICES ON 1ST EFFOFT-HAS TO CHOOSE AGAIN TO GET THE ITEM TO 'STAY'		
		WOULD LIKE TO SEE MORE THAN PAST WEEK FOR THE "FRONT PAGE" TO BE ABLE TO COMPARE ALL	

		OF HIS DATA TO CURRENT WEEK	
SIMPLE, EASY TO USE. HAS NOT YET READ SLEEP TACTICS	EASY TO MAKE RESPONSES. UNABLE TO ADD ADDITIONAL INFO(I.E. NOTES)		
	SHOULD HAVE A WRITE-IN SPACE FOR WASO REASONS (PRIMARY REASON FOR WAKING AT NIGHT IS NOT IN DROP-DOWN MENU)		
	DISCOVERED THAT HE CAN ALTER COMPLETED SLEEP LOGS BY …[DID NOT FINISH]		
DID READ SLEEP TACTICS. FOUN THE LEVEL OF DETAIL TO BE USEFUL. USED THE SHORT BULLET POINTS FOR TROUBLE-SHOOTING AND THE DETAIL FOR MORE BACKGROUND	SIMPLE, CUT, AND DRY		
SUBJECT REPORTED NO COMMENTS OR SUGESSTIONS			
	SCREEN MOVED UPWARD WHEN TRYING TO ENTER TOTAL WASO, WAS STILL ABLE TO ENTER THE MINUTES		
WE REVIEWED THE MATERIAL FIRST IN-SESSION, THEN HE USED APP AS REVIEW			STEP 4:(SUDDEN APPEARANCE OF STRANGE/FOREIGN LANGUAGE) ON SAMSUNG(PLATFORM)-LET WAYAN KNOWN ABOUT CHARACTERS AND WORDS IN FOREIGN LANGUAGE
	PROVIDE OPTION TO SELECT FINAL WAKE UP FROM BATHROOM BREAK		
HAVING TO ENTER DATA CUMBERSTOME. DIDN'T WANT TO HAVE TO LOOK AT CLOCK			
WOULD LIKE IF UNIT COULD DETERMINE TIME AWAKE AT NIGHT AND RETURN TO SLEEP			
	REQUIREMENT TO BE COGNIZANT OF TOTAL MINUTES AWAKE DURING NIGHT DISTURBED EFFORTS TO RESUME SLEEP	ADD ABILITY FOR SYSTEM TO RECOGNIZE AND CALCULATE PERIODS OF SLEEP AND WAKE DURING THE NIGHT!!!	AUTOMATING ANSWERS TO THE MANY QUESTIONS EACH MORNING AND NIGHT WOULD MAKE THE SYSTEM FAR MORE CONVENIENT AND

			ENJOYABLE TO USE.
DISCREPANCY BETWEEN DATA BEING ENTERED ON APP AND THE DATA THAT IS DOWNLOADED FROM FITBIT IS PROBLEMATIC, CAUSING PARTICIPANT TO QUESTION UTILITY AND/OR VALIDITY OF THE DATA			
IF IT WORKED BETTER WITH COMMUNICATION WITH FITBIT AND IREST WOULD BE GOOD			
	2 OR 3 DAYS IN A ROW WOULDN'T LET ME INPUT DATA, AND IT KEPT THE FIELDS POPULATED WITH THE LAST DATA ENTERED	IT WOULD BE NICE TO HAVE THE FITBIT TALK DIRECTLY WITH THE APP AND FACTOR IN "WENT TO BED" TIME W/SLEEP ON FITBIT	
THE APP NEEDS NO IMPROVEMENTS	WORKS FINE	WORKS FINE	WORKS FINE
IT'S A WONDERFUL APP AND WOULD BE HELPFUL FOR ANYONE			
GREAT SYSTEM EASY TO USE AND MONITOR SLEEP EASY	REAL EASY TO USE. VERY SIMPLE. BELIEVE ALL PEOPLE OF ALMOST ALL AGES CAN LEARN TO USE IT NO PROBLEM.	THINK IT WOULD HELP IF THERE WAS A SEPARATE JOURNAL TO WRITE ABOUT YOUR SLEEP IN YOUR OWN WORDS.	
EASY TO USE			
BUG: SLEEP DISTURBANCES DROP DOWN MENU -OFTEN HAVE TO ENTER "TIMES USED BATHROOM" ATTEMPTS >3X	SEE ABOVE		
